# Langerhans cell histiocytosis of the jaw: clinical analysis of 68 cases

**DOI:** 10.1186/s13023-025-03680-w

**Published:** 2025-04-21

**Authors:** Jiale Li, Hao Wu, Zilin Wang, Jing Han, Jiannan Liu, Bing Han

**Affiliations:** 1 Department of Oral and Maxillofacial Surgery, SchoolandHospitalofStomatology, Jilin University, No. 1500, Qing Hua Road, Changchun, 130021 China; 2 Department of Oral and Maxillofacial Head and Neck Oncology, Shanghai Ninth People’s Hospital, Shanghai Jiao Tong University School of Medicine, No. 639, Zhi Zao Ju Road, Shanghai, 200011 China; 3 College of Stomatology, Shanghai Jiao Tong University, Shanghai, China; 4 National Center for Stomatology, Shanghai, China; 5 National Clinical Research Center for Oral Diseases, Shanghai, China; 6 Shanghai Key Laboratory of Stomatology, Shanghai, China; 7 Shanghai Research Institute of Stomatology, Shanghai, China

**Keywords:** Langerhans cell histiocytosis, Jaw, Eosinophilic granuloma, Hand–Schüller–Christian disease

## Abstract

**Background:**

This study aims to investigate the clinical characteristics, imaging features, treatment, and prognostic factors of jaw Langerhans cell histiocytosis (JLCH), providing valuable insights for its clinical diagnosis and management.

**Method:**

This study retrospectively analyzed the clinical and follow-up data of JLCH patients treated between January 2010 and January 2024. Data collected included gender, age, symptoms, imaging findings, treatment strategies, and outcomes. Univariate and multivariate Cox regression analyses were performed using SAS software to identify factors affecting treatment outcomes, with *P* ≤ 0.05 considered statistically significant.

**Results:**

A total of 68 patients (50 males, 18 females; median age 13.5 years) were included. Forty percent of patients were under 10 years old, and 71% had mandibular involvement. Disease classification included 49 cases of single-system unifocal (SS-s) disease, 10 cases of single-system multifocal (SS-m) disease, and 9 cases of multi-system (MS) disease. Common symptoms included jaw or tooth pain (28 cases), facial swelling (22), gingival ulceration (10), and loose teeth (9). Imaging revealed periodontal disease-like (7), cyst-like (17), and osteomyelitis-like (44) lesions. Univariate and multivariate Cox regression analyses identified that female patients had a lower risk of progression (P = 0.014, HR 0.071), while SS-m (P = 0.019, HR 4.992) and MS patients (P = 0.030, HR 4.182) exhibited higher progression risks compared to SS-s patients. Cyst-like (P = 0.001, HR 0.054) and osteomyelitis-like lesions (P < 0.001, HR 0.023) were associated with lower progression risks compared to alveolar lesions.

**Conclusion:**

JLCH can affect individuals of all ages, though it is more common in children. Factors such as gender, lesion multiplicity, and lesion type (alveolar) are significant in predicting disease progression. Complete surgical resection combined with radiotherapy offers the highest likelihood of cure for SS-type JLCH.

## Background

Langerhans cell histiocytosis (LCH) is a rare, etiology-unknown histiocytic disorder with diverse clinical manifestations, often affecting children [1]. It is characterized by the proliferation of CD1a+/CD207+ Langerhans cells and widespread involvement of multiple organs [2]. Langerhans cells were first identified by Paul Langerhans in the mid-nineteenth century and named after him. In 1953, Lichtenstein referred to the condition as “histiocytosis X.” Later, in 1973, Neelov proposed renaming the disease “Langerhans cell histiocytosis” after recognizing that the abnormal cells in histiocytosis X and normal Langerhans cells shared similar ultrastructural features [3].

LCH can affect any organ, with common sites including bones, skin, lymph nodes, liver, spleen, mucosa, lungs, and the central nervous system (CNS) [4]. According to the WHO 2022 clinical classification, LCH is divided into single-system, single-organ LCH (SS-s), single-system, multiple-organ LCH (SS-m), and multi-system LCH (MS-LCH), based on the number of affected systems [5]. SS-s refers to involvement of a single organ or a few sites within a single system (such as bones, skin, lymph nodes, or lungs). MS-LCH involves multiple systems, including potentially risk organs like the liver, spleen, bone marrow, or hematopoietic systems. Lesions in the temporal bone, sphenoid bone, orbit, or mastoid process may indicate CNS involvement, posing a CNS risk [6].

LCH is clinically classified into three types based on presentation: chronic localized (eosinophilic granuloma), chronic disseminated (Hand–Schüller–Christian disease), and acute disseminated (Letterer–Siwe disease) [7]. The chronic localized form manifests as isolated eosinophilic granulomas in the jaw, with little surrounding tissue infiltration and no extraosseous symptoms. The chronic disseminated form (Hand–Schüller–Christian disease) involves multiple bones in the maxillofacial region or soft tissues outside the bone, with clear extraosseous symptoms, including potential visceral involvement (e.g. liver, spleen, lungs, skin, and lymph nodes). It is often associated with the classic triad of skull defects, diabetes insipidus, and exophthalmos. The acute disseminated form (Letterer–Siwe disease) presents with persistent fever, rash, and “floating teeth,” and involves widespread visceral systems, often with a rapid onset and poor prognosis [2, 8].

Bones are the most commonly affected organs in LCH, with flat bones being particularly susceptible, followed by the jaw [9]. JLCH involves the jaw and can either be part of a systemic disease or occur as a localized condition. Studies have indicated that patients with LCH often first present with jawbone symptoms, making oral specialists the first line of defense in diagnosing the condition [10]. Due to its rarity and similarity to other jaw-related diseases [3], LCH is often misdiagnosed or missed in clinical practice [11]. Early diagnosis is crucial for proper treatment and prognosis. JLCH frequently leads to maxillofacial defects, which have significant effects on patients due to delayed diagnosis, dysfunction, and other symptoms. Moreover, there is no clear treatment protocol for JLCH.

This article reviews the medical records of 68 patients diagnosed with JLCH, in conjunction with the existing literature, to analyze their clinical manifestations, differential diagnoses, treatment options, and prognosis. The aim is to summarize their clinical characteristics and disease features, offering insights for clinical diagnosis and management.

## Methods

### Research subjects

This study collected cases of JLCH treated by the Department of Oral and Maxillofacial Surgery, the Department of Oral and Maxillofacial–Head and Neck Oncology, and the Department of Hematology at the Ninth People’s Hospital affiliated with Shanghai Jiao Tong University School of Medicine. The colletion period spanned from January 2010 to January 2024 for retrospective follow-up.

Inclusion criteria were as follows: (1) The histopathological diagnosis of each case was LCH; (2) Lesions occurring within the jaw, with or without involvement of other systems. Exclusion criteria were: (1) Lesions occurring in the maxillofacial region without involving the jaw; (2) Patients diagnosed but unable to obtain follow-up information. According to the inclusion criteria, follow-up information was obtained for 68 individuals, including 50 males and 18 females. All cases were pathologically confirmed and supported by complete medical histories and imaging data. The follow-up period ranged from 0.5 to 14 years, with an average follow-up of 6.83 ± 3.44 years. This study has been approved by the ethics review board of the Ninth People’s Hospital, Shanghai Jiao Tong University School of Medicine.

### Follow-up data

The medical histories of all patients were reviewed to gather clinical data, including age, gender, lesion location, accompanying symptoms, treatment methods, and prognosis. Imaging data, including maxillofacial CT scans, CT scans of the limbs and trunk, MRI scans, X-rays (such as panoramic views), and PET-CT scans, were also examined.

### Diagnostic criteria

According to the 2011 guidelines of the International Histiocyte Society, a diagnosis is established when Langerhans cells proliferate under light microscopy if one of the following three criteria is met: (1) Positive immunohistochemical staining for Langerin (CD207); (2) Positive immunohistochemical staining for CD1a antigen; (3) Presence of Birbeck granules in pathological cells observed via electron microscopy [12, 13]. In this study, all cases underwent pathological and immunohistochemical testing, confirming positivity for S-100, CD1a, and Langerin. Birbeck granules were detected in the cytoplasm of tissue cells under electron microscopy, all of which were confirmed by pathological diagnosis.

### Research methods

A retrospective case study approach was used to analyze the incidence, clinical manifestations, imaging findings, treatment methods, and prognosis of JLCH. Patients were categorized based on age (≤ 10 years, > 10 and < 18 years, ≥ 18 years), imaging classification (alveolar lesion type, intraosseous jaw lesion type—cystoid and osteomyelitis types), affected site types (SS-s, SS-m, MS), soft tissue involvement, treatment modalities (simple curettage, curettage with chemotherapy/radiotherapy, segmental resection of the jaw, segmental resection with chemotherapy/radiotherapy, biopsy with chemotherapy, biopsy with chemotherapy/radiotherapy), and prognosis (cured, stable, progressive).

Prognosis evaluation followed the LCH therapeutic efficacy criteria set by the International Histiocyte Society in 1991, which include: Cured—symptoms and/or objective signs disappear with no new lesions; Stable—symptoms and/or objective signs persist without new lesions; Progressing or worsening—symptoms and/or objective signs worsen compared to baseline, and new lesions appear or old lesions recur.

All data were managed and analyzed using SAS 9.4 (version 9.4 for Windows, SAS Institute, Inc., Cary, NC, USA). Qualitative data were presented as n (%). Univariate and multivariate Cox regression analyses were performed to assess factors affecting cure, and Kaplan–Meier curves were generated. Statistical significance was set at P ≤ 0.05.

## Results

### Age and gender distribution of the disease

**Fig. 1:**
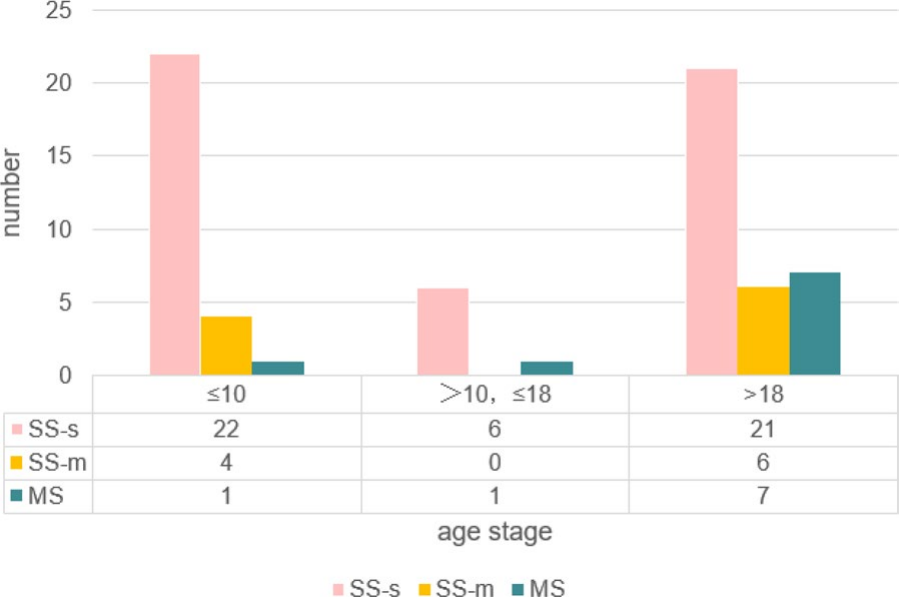
Age distribution of the three classes of patients

This cohort consisted of 68 patients, including 50 males and 18 females. The age range was from 1 month to 62 years, with a median age of 13.5 years. Forty-nine patients were diagnosed with chronic localized JLCH (SS-s), characterized by localized eosinophilic granuloma of the jaw without significant surrounding tissue infiltration. Nineteen patients were classified as chronic disseminated type, including 10 with SS-m and 9 with MS (Hand–Schüller–Christian disease). No cases of acute disseminated type (Letterer–Siwe disease) were observed. The distribution of gender and age across these types is summarized in Table 1. Across all JLCH types, males outnumbered females. Figure 1 shows the age distribution for each type: SS-s occurred in all age groups, SS-m was absent in the 10–18 age group, and MS cases were most common among adults.

**Table 1:** Gender and age distribution of the 68 patients

	Cases	Gender		Age		
		Males	Females	Oldest	Youngest	Median age
SS-s	49	38	11	62	1	14
SS-m	10	7	3	52	3.5	19
MS	9	5	4	54	4	20.5

### Lesion sites and clinical features

Among the 68 patients in this cohort, 53 cases (77.94%) involved the unilateral mandible, 3 cases affected both sides of the mandible, 5 cases involved both the maxilla and mandible simultaneously, and 7 cases were restricted to the maxilla. Twenty-eight patients primarily complained of jaw swelling, pain, or discomfort, while 22 patients reported swelling and pain in the maxillofacial region. Ten patients presented with gingival tumors and erosive destruction along the gingival margin resembling caterpillar tracks, 9 experienced gingival swelling, tooth loosening, and tooth loss, and 2 cases involved soft tissue extending to the facial skin. Four cases showed temporal swelling and temporal bone destruction, 11 cases had concurrent bone destruction in other areas (e.g., cranial vault, vertebrae, ribs), and 9 cases exhibited symptoms in other systems (e.g., diabetes insipidus, liver damage). The distribution of lesion sites and clinical features across the three patient types is summarized in Table 2.

**Table 2:** Summary of lesion sites and clinical features of the 68 patients

Characteristic	SS-s	SS-m	MS	Total
*Position*				
SS-s mandible	44	6	3	53
SS-s maxilla	5	1	1	7
SS-m mandible	–	1	2	3
SS-m mandible and maxilla	–	2	3	5
*Clinical manifestation*				
Gingival swelling and pain/ulceration/mass	8	–	2	10
Teeth loose/loss	4	2	3	9
Maxillofacial swelling and pain	16	4	2	22
Involves the skin/skin breakdown	1	1	–	2
Jaw swelling/pain	25	3	1	28
Lower lip numbness	–	1	–	1
Limitation of mouth opening	5	–	–	5
With other bone destruction	–	7	8	15
With other systemic diseases (Abnormal pituitary function, diabetes insipidus, liver injury)	–	–	9	9

### Imaging features of jaw lesions

**Fig. 2:**
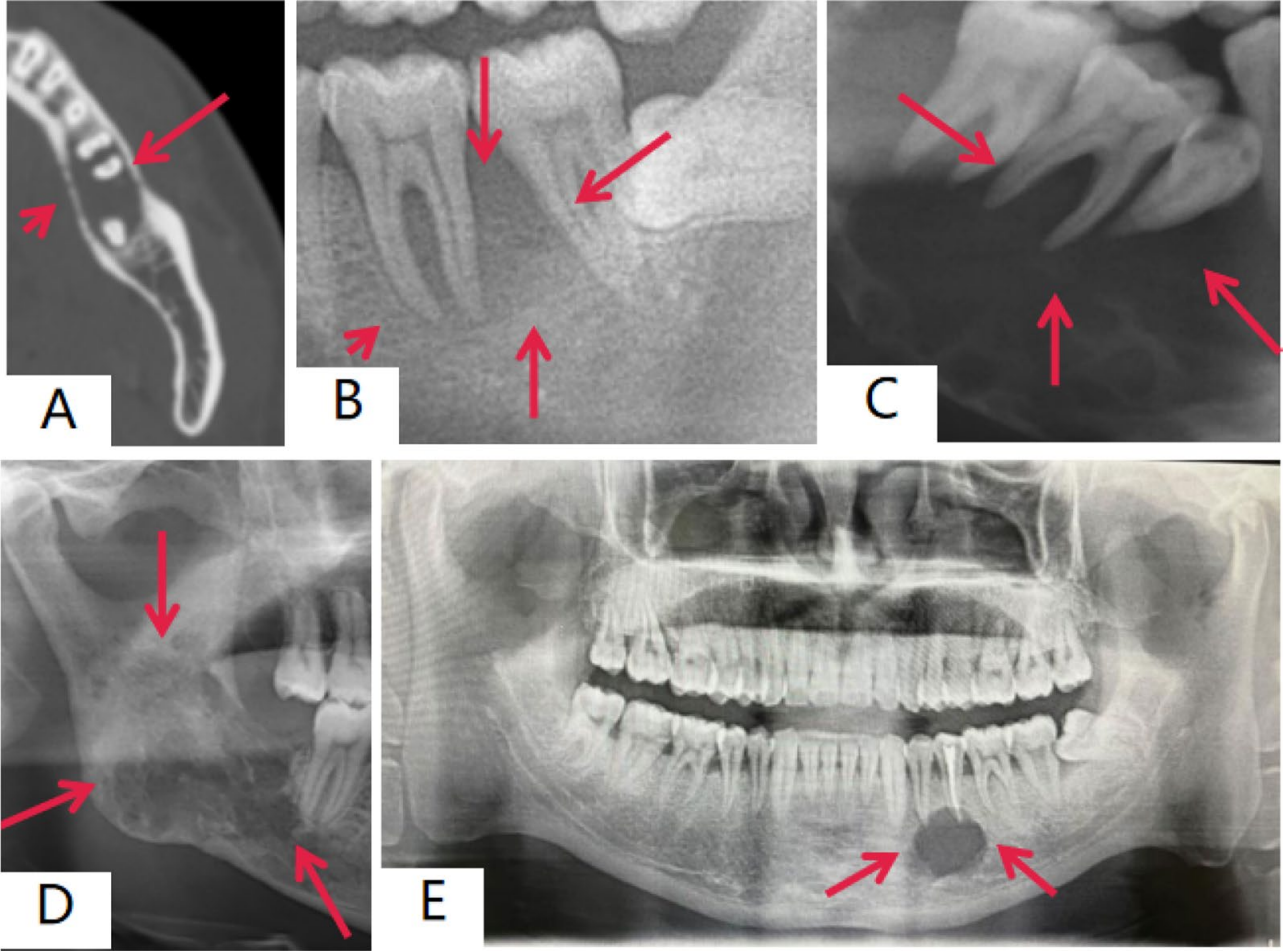
Imaging manifestations of different types of JLCH. **A**, **B** Alveolar lesion type; **C** Floating tooth appearance; **D** Intraosseous jaw lesion type—osteomyelitis type; **E** Intraosseous jaw lesion type—cystoid type

JLCH primarily presents with erosive bone destruction and soft tissue involvement. Among the 68 patients, all had lesions in the jaw, with 25 cases also involving soft tissues. On coronal CT scans, JLCH typically appears as irregular bone destruction with variable border clarity, resembling lytic lesions. When teeth are affected, there is usually periapical low-density shadowing, without signs of resorption. In some cases, a “floating tooth” appearance (Fig. 2C) can be observed, where the tooth itself appears unaffected, but surrounding tissues exhibit pathology, giving the illusion that the tooth is floating above the jaw.

CT and MRI scans of the maxillofacial region reveal bone destruction in the jaw with cortical swelling on both the buccal and lingual sides, often accompanied by cortical bone destruction and even pathological fractures, with or without soft tissue involvement. Among the 68 patients, 7 exhibited bone resorption around the tooth roots (1 case showed horizontal absorption of alveolar bone due to periodontal disease, Fig. 2AB). Seventeen cases displayed single or multilocular cyst-like changes with clear boundaries (Fig. 2E). Forty-four cases showed blurred trabecular bone structure with osteolytic destruction, similar to osteomyelitis (Fig. 2D). Additionally, 22 cases exhibited periosteal reactions, and 3 cases had continuous bone interruptions.

### Treatment and prognosis

**Fig. 3:**
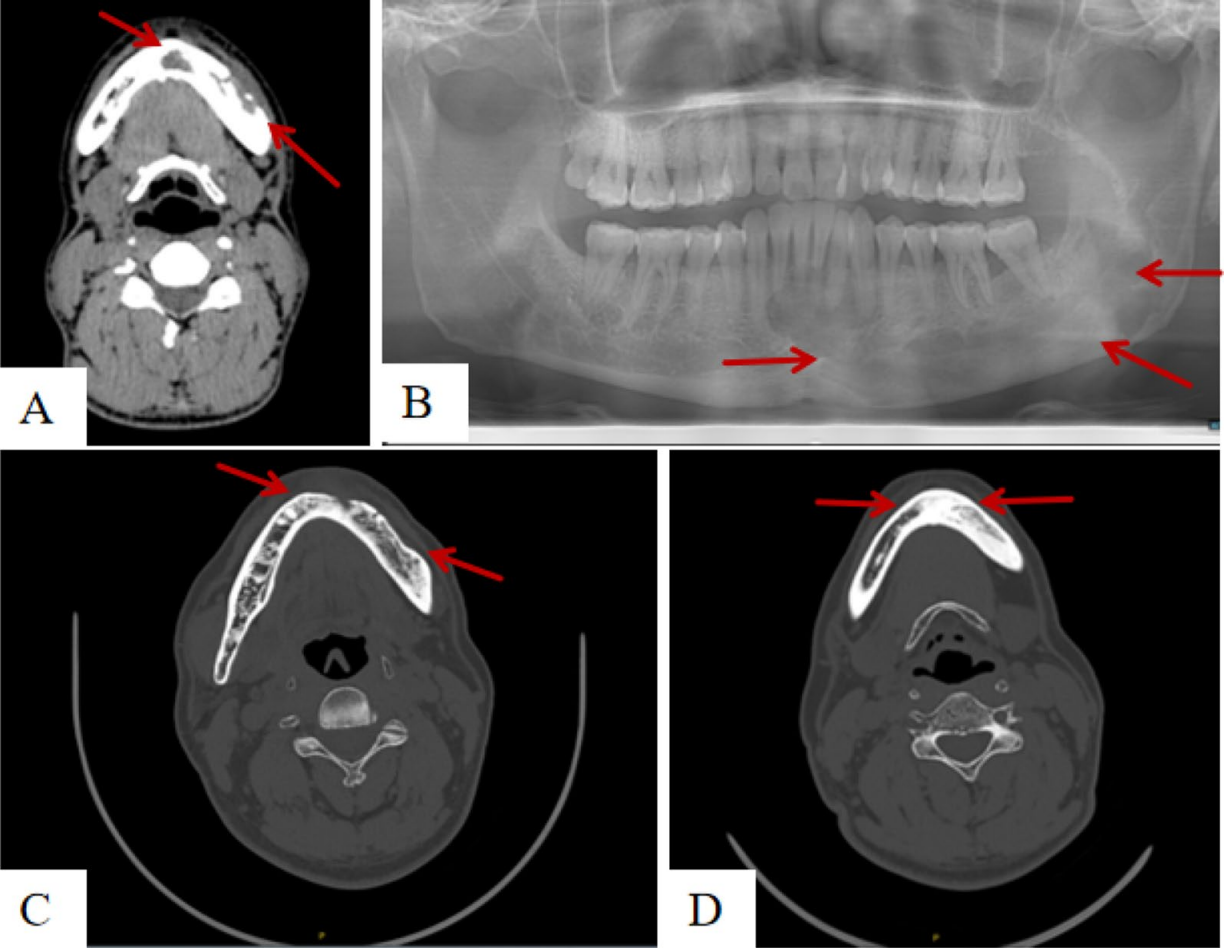
Preoperative and postoperative imaging of a case of mandibular LCH treated with curettage combined with radiotherapy. **A** CT shows a lesion in the left mandible with unclear borders and partial loss of buccal cortical bone. **B** Panoramic tomography shows a well-defined but heterogeneous low-density shadow in the body and ramus of the left mandible. **C**, **D** CT images taken 4 years post-curettage and radiotherapy show a reduced lesion size with visible bone repair at the edges and no recurrence

**Fig. 4:**
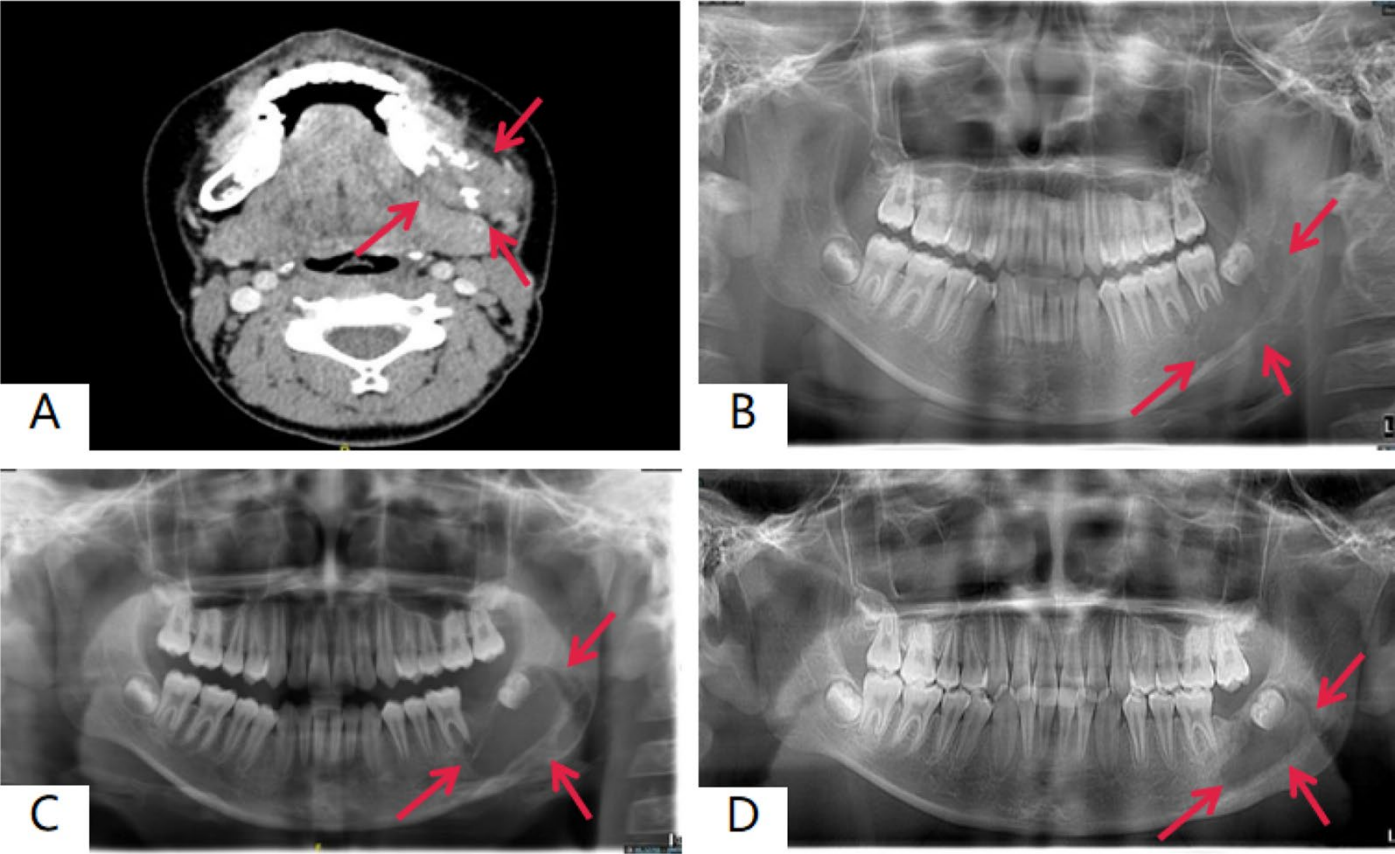
Preoperative and postoperative imaging of a case of mandibular LCH treated with biopsy combined with chemotherapy. **A** CT shows a lesion in the left mandible with unclear borders and lytic destruction involving the buccal soft tissue. **B** Panoramic tomography shows a low-density shadow with unclear borders in the body and ramus of the left mandible. **C**, **D** Post-chemotherapy CT shows a reduction in the lesion size with visible bone repair at the edges and no recurrence

Treatment for all 68 patients, analyzed by type (SS-s, SS-m, MS), involved a range of therapeutic approaches, as detailed in Tables 3, 4, and 5. Three patients with type SS-s were lost to follow-up. Among the treatments, 17 cases underwent simple curettage (SC), resulting in 11 cures and 4 cases of progression. Five patients received curettage combined with radiotherapy (C + RT) (Fig. 3), with 4 cured and 1 stable. Another five cases underwent curettage combined with chemotherapy (C + Chemo), yielding 2 cures, 1 stable, and 2 progressions. Six cases underwent biopsy combined with radio and chemotherapy (B + Chemo + RT) (Fig. 4), with 1 cured and 2 progressing. Eleven patients required segmental resection of the mandible with tissue flap repair or joint replacement due to mandibular discontinuity (SR + TF), extensive soft tissue involvement, or joint damage, with no cases of progression. Finally, two patients underwent complete excision of the lesion followed by flap repair and radiotherapy/chemotherapy (SR + TF + RT/Chemo), both showing disease progression. No patient died during the study.

**Table 3:** Different treatment regimens for 46 cases of SS-s type patients

	Cured	Stable	Progressing
SC	11	–	2
C + Chemo	2	1	1
C + RT	3	1	–
SR + TF	9	–	–
SR + TF + RT/Chemo	–	–	2
B + Chemo	5	5	2
B + Chemo + RT	1	–	1

*SC* Simple curettage, *C + Chemo* Curettage + chemotherapy, *C + RT* Curettage + radiotherapy, *SR + TF* Segmental resection with tissue flap, *SR + TF + RT/Chemo* Segmental resection with tissue flap + radiotherapy/chemotherapy, *B + Chemo* Biopsy + chemotherapy, *B + Chemo + RT* Biopsy + chemotherapy and radiotherapy

**Table 4:** Different treatment regimens for 10 cases of SS-m type patients

	Cured	Stable	Progressing
SC	–	–	2
C + Chemo	–	–	1
C + RT	1	–	–
SR + TF	2	–	–
B + Chemo	–	2	1
B + Chemo + RT	–	1	–

**Table 5:** Different treatment regimens for 9 cases of MS type patients

	Cured	Stable	Progressing
B + Chemo	–	3	3
B + Chemo + RT	–	2	1

### Survival analysis results

**Fig. 5:**
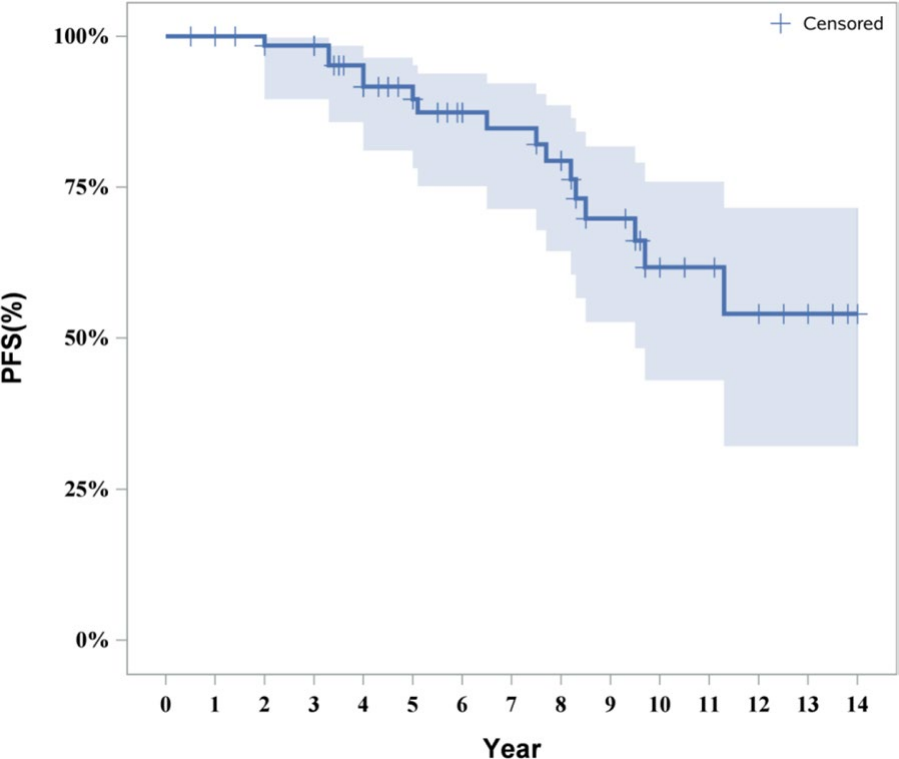
Progression-free survival curve

Sixty-eight subjects were followed up, with baseline information shown in Table 6. The median follow-up time was 6 years (range 0.5–14 years). The 3-year progression-free survival (PFS) rate was 95.18%, the 6-year PFS rate was 84.74%, and the 12-year PFS rate was 54.00%. Detailed information is provided in Fig. 5.

**Table 6:** Baseline characteristics of the study participants [n (%)]

Variable	Group	Description
Gender [*n*(%)]	Male	50 (73.53)
	Female	18 (26.47)
Age of onset ($\overline{x} \pm s$)		22.27 ± 18.76
Age of onset [*n*(%)]	≤ 10	27 (39.71)
	> 10, ≤ 18	7 (10.29)
	> 18	34 (50.00)
Position [*n*(%)]	SS-s	49 (72.06)
	SS-s	10 (14.71)
	SM	9 (13.24)
Imaging findings [*n*(%)]	Alveolar lesion type	7 (10.29)
	Cystoid type	17 (25.00)
	Osteomyelitis-like	44 (64.71)
Other systems [*n*(%)]	With	43 (63.24)
	Without	25 (36.76)
Treatment method [*n*(%)]	SC	17 (25.00)
	SR + TF	11 (16.18)
	C + Chemo/RT	10 (14.71)
	SR + TF + RT/Chemo	2 (2.94)
	B + Chemo	22 (32.35)
	B + Chemo + RT	6 (8.82)

Univariate Cox analysis results showed that adult patients had a higher likelihood of progression compared to pediatric patients (HR 3.55, 95% CI 1.13–11.21). Patients with MS JLCH involvement had a greater risk of progression compared to those with SS involvement (HR 3.40, 95% CI 1.02–11.32). Among the imaging features of intraosseous lesions, the risk of progression for cyst-like (HR 0.09, 95% CI 0.02–0.52) and osteomyelitis-like (HR 0.05, 95% CI 0.01–0.26) lesions was lower than that for maxillary bone protuberance destruction. Detailed information is provided in Table 7.

**Table 7:** Single-factor cox analysis

Variable	Number of observations	Number of events	Rate(1/100 ppl*)	*P*	HR (95%CI)
*Gender*					
Male	50	15	4.536		1.00 (ref)
Female	18	1	0.746	0.058	0.14 (0.02–1.07)
*Age of onset*				0.067	1.03 (1.00–1.06)
*Age of onset*					
≤ 10	27	4	1.867		1.00 (ref)
> 10, ≤ 18	7			0.994	0.00 (0.00–)
> 18	34	12	5.712	**0.030**	3.55 (1.13–11.21)
*Position*					
SS-s	49	8	2.349		1.00 (ref)
SS-s	10	4	5.510	0.210	2.16 (0.65–7.20)
SM	9	4	7.767	**0.046**	3.40 (1.02–11.32)
*Imaging findings*					
Alveolar lesion	7	3	14.085		1.00 (ref)
Cyst-like	17	6	4.594	**0.006**	0.09 (0.02–0.52)
Osteomyelitis-like	44	7	2.238	** < 0.001**	0.05 (0.01–0.26)
*Soft tissue involvement*					
With	43	12	3.828		1.00 (ref)
Without	25	4	2.646	0.617	0.75 (0.24–2.33)
*Treatment method*					
SC	17	4	3.205		1.00 (ref)
SR + TF	11			0.992	0.00 (0.00–)
C + Chemo/RT	10	2	2.320	0.752	0.76 (0.14–4.16)
SR + TF + RT/Chemo	2	2	11.628	0.150	3.51 (0.63–19.40)
B + Chemo	22	6	4.249	0.519	1.52 (0.43–5.40)
B + Chemo + RT	6	2	6.270	0.277	2.60 (0.46–14.54)

**Fig. 6:**
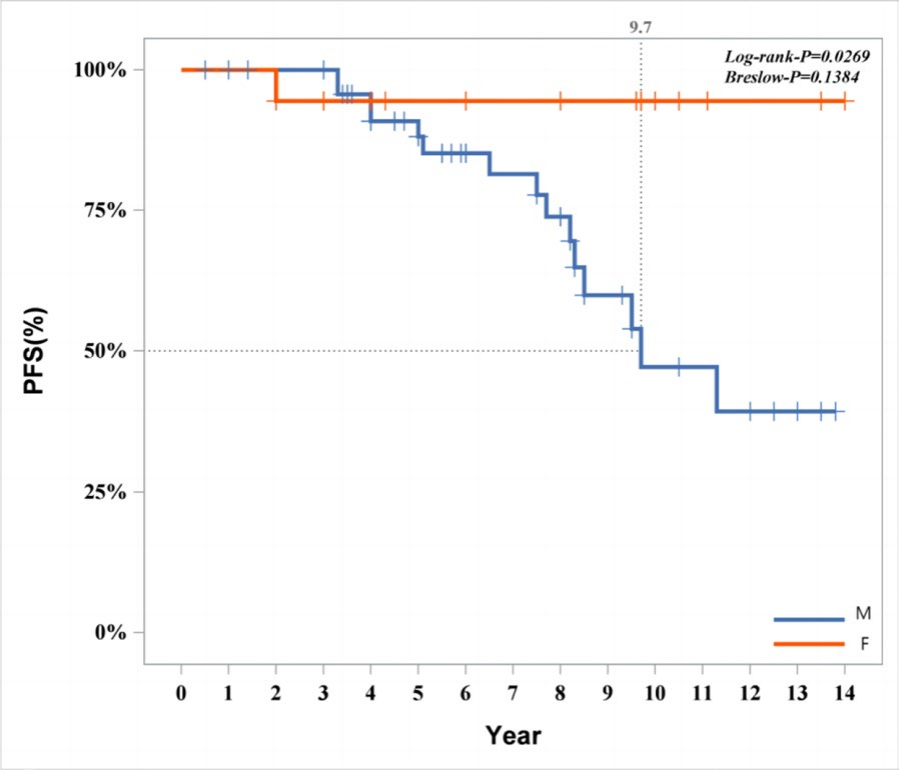
Progression of patients of different gender

**Fig. 7:**
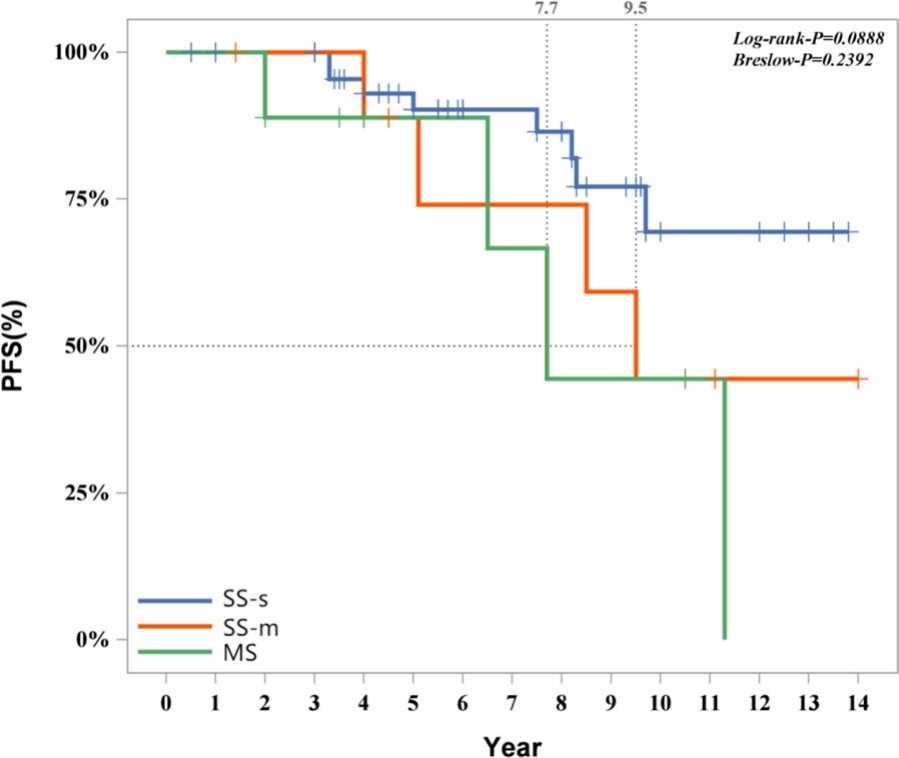
Cure of patients with different lesions

**Fig. 8:**
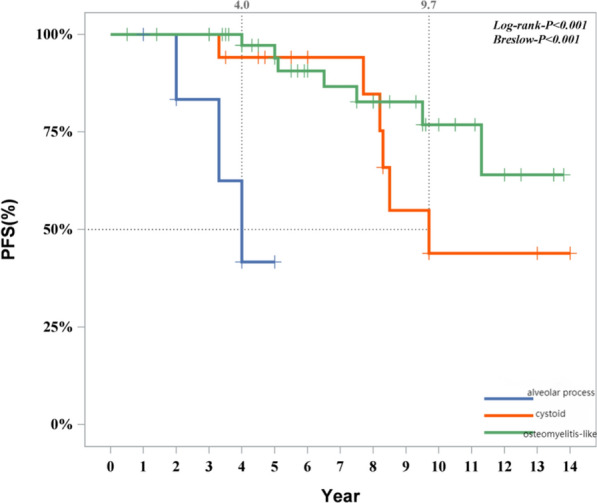
Progression of patients with different imaging findings

The following cure outcomes were taken as the dependent variable; univariate significant variables were included as independent variables (categorical variables were incorporated as dummy variables). A stepwise method was used to fit the multivariate Cox regression model. The model fitting results indicated that gender, location (whether multisystem or multifocal), and imaging features (whether alveolar lesion type) were influencing factors for disease progression. Female patients had a lower likelihood of progression compared to male patients (*P* = 0.014, HR 0.071, 95% CI 0.009–0.596). Detailed information is provided in Fig. 6. Patients with SS-m (*P* = 0.019, HR 4.992, 95% CI 1.291–19.300) and those with MS (*P* = 0.030, HR 4.182, 95% CI 1.145–15.268) had a higher likelihood of progression compared to patients with SS-s (Fig. 7). Among intraosseous lesion types, the risk of progression for cyst-like lesions (*P* = 0.001, HR 0.054, 95% CI 0.009–0.341) and osteomyelitis-like lesions (*P* < 0.001, HR 0.023, 95% CI 0.004–0.144) was lower than that for alveolar lesion type (Fig. 8). Detailed information is provided in Table 8.

**Table 8:** Multivariate-factor cox analysis

Variable	Estimation	Standard Error	Chi-Square Value (χ^2^)	*P*	HR (95%CI)
*Gender*					
Male					1.00 (ref)
Female	− 2.642	1.084	5.943	**0.014**	0.071 (0.009–0.596)
*Position*					
SS-s					1.00 (ref)
SS-s	1.608	0.690	5.430	**0.019**	4.992 (1.291–19.300)
SM	1.431	0.661	4.689	**0.030**	4.182 (1.145–15.268)
*Imaging findings*					
Alveolar lesion type					1.00 (ref)
Cyst-like	− 2.914	0.938	9.649	**0.001**	0.054 (0.009–0.341)
Osteomyelitis-like	− 3.781	0.940	16.173	** < 0.001**	0.023 (0.004–0.144)

## Discussion

Langerhans cell histiocytosis (LCH) is a benign yet aggressive disease that presents with a variety of clinical manifestations. Previous studies report an incidence of 1–2 cases per million in children, which increases to 3-5 cases per million, with a peak incidence between ages 3 and 4. The male-to-female ratio is approximately 2:1 [14]. Jaw LCH (JLCH) primarily affects individuals under 15 years of age [15], with 90% of cases involving the head and neck region, particularly the skull and jaw, with a greater prevalence in the mandible compared to the maxilla [9, 16–18]. In the current study, the patient cohort consisted of 50 males and 18 females, reflecting a male predominance consistent with existing literature. The age distribution demonstrated a median onset of 13.5 years, with 47.06% of patients being younger than 15 years, including nearly 40% under the age of 10, suggesting a higher prevalence in children. Notably, a higher proportion of SS-s patients were under 10 years old, while MS patients were predominantly adults, indicating that age may significantly influence the prognosis of JLCH.

Radiographically, JLCH manifests in three distinct types: periodontitis-like, cyst-like, and osteomyelitis-like, with a primary feature of lytic bone destruction [19]. The periodontitis-like type typically affects the alveolar ridge and presents symptoms such as tooth pain, mobility, loss, gingival hyperplasia, and ulceration, resembling severe periodontitis, periapical periodontitis, or malignancy [9]. In this study, seven patients with alveolar ridge lesions exhibited tooth mobility or alveolar bone destruction at the mid-root level [20], mimicking severe periodontitis but without obvious carious teeth. The cyst-like and osteomyelitis-like types generally affect the jaw body, with 61 patients experiencing jaw and/or facial swelling and pain. Severe cases presented with skin ulceration and restricted mouth opening when lesions involved the mandibular angle or ramus. Radiographic findings included clear, cyst-like lesions or more indistinct osteomyelitis-like features, with osteolytic destruction, jaw expansion, surrounding sclerosis, periosteal reaction, and pathological fractures [21]. About 25% of patients exhibited well-defined, round low-density lesions, while 64.71% demonstrated diffuse lytic destruction. Notably, no neurological symptoms were observed, even in cases with nerve canal compression or mandibular continuity disruption. Additionally, 36% of patients with intraosseous lesions showed periosteal reactions, distinguishing them from osteomyelitis, which is more common in adults. In the absence of significant inflammatory stimulation, unilateral or bilateral osteolytic lesions can often be diagnosed clinically, and treatment plans can be tailored after biopsy. Therefore, radiographic classification is crucial for the clinical diagnosis and early management of JLCH.

The definitive diagnosis of LCH is based on histopathological examination, and treatment decisions depend on factors such as lesion location, size, and symptoms. Treatment guidelines advocate for both local and systemic therapies [22]. Local treatments include surgical excision, intralesional corticosteroid injections, radiotherapy, and medical therapy. Literature suggests that local therapies are effective for single-system involvement, while systemic therapy is recommended for multi-system involvement [23]. In this study, 22 SS-s patients treated with curettage alone had a favorable outcome, with only 2 cases of disease progression, indicating that complete surgical excision is effective for most patients. Among the 4 patients treated with curettage combined with radiotherapy, all were cured, demonstrating excellent outcomes. Five patients treated with curettage combined with chemotherapy had mixed results, with 2 cures, 1 stable case, and 2 cases of disease progression. These findings suggest that while curettage alone is effective for some SS-s patients, combining curettage with radiotherapy may reduce the risk of progression.

For SS-m patients, the study included 10 cases treated with varying approaches. One patient was cured with curettage and radiotherapy, while two were cured with segmental resection and tissue flap reconstruction. However, other treatments, including curettage combined with chemotherapy and biopsy combined with radiochemotherapy, resulted in disease progression. These results highlight that multi-lesion patients typically require a combination of treatments, with radiotherapy potentially reducing the risk of CNS involvement. For MS patients, our study included 9 cases treated with biopsy combined with chemotherapy or radiochemotherapy, none of which resulted in a cure, and 4 cases showed progression. These findings suggest that surgical excision combined with radiotherapy or medical therapy may still represent the best treatment approach.

Based on the existing literature and the results from this study [24–26], we propose the following hypotheses: Curettage alone is suitable for SS-s patients with localized lesions and minimal soft tissue infiltration. For patients with larger lesions, pathological fractures, or functional impairment, segmental mandibulectomy with tissue flap reconstruction followed by implant restoration can restore facial appearance and occlusal function with good long-term prognosis. Curettage combined with radiotherapy is recommended for SS-s patients with larger lesions or partial soft tissue involvement. For SS-m patients with skull involvement, excision of jaw lesions combined with radiotherapy or chemotherapy can reduce CNS risks. According to the 2013 treatment guidelines for children under 18, single-system or low-risk LCH in children typically involves local treatments, such as curettage or low-dose radiotherapy [27], which aligns with our findings in JLCH.

While the present study contributes valuable insights, it has limitations, including a small sample size, short follow-up duration, and a lack of multi-center randomized controlled trials. Additionally, the long-term effects and potential complications of various treatments warrant further investigation. Future studies should focus on large, multi-center trials to validate the efficacy and safety of treatment options, as well as long-term follow-up to evaluate outcomes and complications. These efforts will provide a stronger foundation for optimizing JLCH treatment and improving patient prognosis.

## Conclusion

Langerhans cell histiocytosis (LCH) remains a relatively rare and complex disease. Despite its distinctive clinical and radiological features, it must be differentiated from several other conditions. Early diagnosis is crucial for reducing LCH-related complications and ensuring a favorable prognosis, particularly in children. Prognosis in JLCH patients is influenced by factors such as gender, lesion sites, and radiological manifestations. Males, patients with multiple lesions, and those with alveolar ridge involvement are at higher risk of disease progression, making early systemic therapy essential. For patients with single-system JLCH, complete surgical resection combined with radiotherapy offers a higher likelihood of cure.

## Data Availability

All data supporting the findings of this study are available within the paper and its Supplementary Information. All data are available from the corresponding author upon reasonable request.

## Abbreviations

LCH: Langerhans cell histiocytosis
JLCH: Langerhans cell histiocytosis of the jaw
SS-s: Single-system unifocal
SS-m: Single-system multifocal
MS: Multi-system
SC: Simple curettage
C + Chemo: Curettage + chemotherapy
C + RT: Curettage + radiotherapy
SR + TF: Segmental resection with tissue flap
SR + TF + RT/Chemo: Segmental resection with tissue flap + radiotherapy/chemotherapy
B + Chemo: Biopsy + chemotherapy
B + Chemo + RT: Biopsy + chemotherapy and radiotherapy
